# Coming New Age of Marine Glycomics: The Fundamental, Medical, and Ecological Aspects

**DOI:** 10.3390/md20100613

**Published:** 2022-09-28

**Authors:** Yuki Fujii, Marco Gerdol, Yasuhiro Ozeki

**Affiliations:** 1 Graduate School of Pharmaceutical Sciences, Nagasaki International University, 2825-7 Huis Ten Bosch, Sasebo 859-3298, Japan; 2 Department of Life Sciences, University of Trieste, Via Licio Giorgieri 5, 34127 Trieste, Italy; 3 Graduate School of NanoBio Sciences, Yokohama City University, 22-2 Seto, Kanazawa-ku, Yokohama 236-0027, Japan

This Special Issue “Marine Glycomics” (https://www.mdpi.com/journal/marinedrugs/special_issues/Marine_Glycomics, accessed on 12 September 2022) provided new approaches and information on bioactive compounds, such as glycans and lectins from marine animals, seaweeds, and microorganisms for the application of clinical therapy and elucidation of the physiological functions of marine organisms.

Marine glycomics is a discipline which concerns the study of glycans and their binding partners, i.e., lectins, in marine species. First, we would like to introduce the articles and reviews related to glycans that were published in this Special Issue.

A review by Fonseca and Mourao discusses the pharmacological activity of sulfated fucose-rich polysaccharides, which can be administered orally [1]. Many reports of polysaccharides’ pharmacological effects were examined using experimental animal models. These polysaccharides are available from echinoderms (sea urchins and sea cucumbers) and algae. This review describes several functions of polysaccharides, such as their antilipidemic, anticancer, and immunomodulatory activities, as well as their effects on diabetes, thrombosis, and hemostasis. Finally, this review summarizes the potential of oral treatment of various diseases by utilizing sulfated polysaccharides.

Lin, Li, Gao et al. introduce the anticoagulation function of fucosylated glycosaminoglycan (FG) derived from two species of sea cucumber, *Thelenota bananas* and *Holothuria fuscopunctata* [2]. Both high and low (depolymerized) molecular weight of FG showed anticoagulation properties. Nevertheless, the injection of high molecular weight FG caused the death of rats due to the induction of hypotension through the activation of rat plasma kallikrein and an increase in the level of the vasoactive peptide bradykinin. On the other hand, low molecular weight FG did not show any side effects. The authors argue that depolymerized FG will be able to specifically target iXase, allowing its use as a safe anticoagulant compared to native FG.

A review by Besednova, Zaporozhets, Andryukov et al. discusses the antiparasitic activity of sulfated polysaccharides (SPS) obtained from marine algae and invertebrates [3]. SPS has potential for the treatment and prevention of infections caused by several types of protozoa, as well as the prevention of helminthiasis. The combination treatment of silver nanoparticles and fucoidans from brown algae showed an antitrypanosomal effect. Some SPS, such as fucoidans or their derivatives, have also reported trypanocidal activity. Cryptosporidiosis and trichomoniasis are also potential therapeutic targets of SPS. These findings will not only contribute to the design of novel SPS-based drugs, but also to the development of biologically active food additives and functional food products with antiparasitic activity.

A review by Qi, Zhou, Shen et al. discusses the considerable potential of bioactive compounds, including peptides and polysaccharides derived from peanut worms (Annelida, Sipuncula), which may find application both in food and pharmaceutical industry [4]. Peanut worms (e.g., *Sipunculus nudus*) are used in traditional Chinese medicine for the treatment of different medical conditions, such as hypertension, neurosis, and cough, and for many other purposes. The 162 known species of peanut worms, all living in shallow water, have a significant potential for the discovery of novel bioactive compounds. Peanut worm-based molecules endowed with antioxidant, anti-inflammatory, antihypertensive, anticancer, and wound healing activities have already been reported. Based on these observations, the authors discuss the great potential that peanut worms might have for the discovery of new peptides and polysaccharides with antidiabetic, antimicrobial, and many other biologically relevant properties.

Ren, Gao, Dai et al. report the bioactivity of light-yellow pigmented bacterial strains, which were newly isolated from the strongly toxic and dinoflagellate *Alexandrium catenella*, associated with harmful algal blooms [5]. Taxonomic and phenotypic analyses allowed the identification of this bacteria as a *Mameliella alba* strain. Nevertheless, the authors found an extraordinary production of bioflocculanting exopolysaccharides. Interestingly, the portion of exopolysaccharides containing glucose and fucose positively contributed to this flocculant ability. These findings revealed the potential pharmaceutical, environmental, and biotechnological implications of active EPS produced by this new *Mameliella alba* strain. It also highlighted the importance of studying bacteria associated with harmful algae due to the presence of unique compounds that still remain to be uncovered. 

Next, we would like to introduce advanced findings published in this species issue related to the identification and functional characterization of marine lectins.

Gerdol provides a first insight into the diversity of secretory glycan-binding lectin gene from Rotifera, a phylum of small aquatic animals belonging to Lophotrochozoa [6]. While a high number of lectins have been described in lophotrochozoans, no information was previously available about rotifer lectins. The author identified secretory lectin-like sequences from the genomes of from six rotifer species, *Brachionus calyciflorus*, *Brachionus plicatilis*, *Proales similis* (class Monogononta), *Adineta ricciae*, *Didymodactylos carnosus,* and *Rotaria sordida* (class Bdelloidea). At least nine lectin families were identified, including fibrinogen-related domain-containing proteins (FreDs), C-type lectins, C1q domain-containing proteins, galectins, R-type lectins, F-type lectins, SUEL-type lectins, H-type lectins, and jacalin-type lectins. Each of the lectin families is characterized by a different structural fold. Interestingly, several unique features were identified in rotifer lectins, supporting the importance of expanding the range of organisms targeted by lectins research in order to elucidate their role in the future.

Takeuchi, Jimbo, Tanimoto et al. describe the symbiosis between the coral *Acropora tenuis* and Symbiodiniaceae cells, discussing the involvement of a coral lectin in this interaction [7]. The coral N-acetyl-d-glucosamine-binding lectin ActL plays a fundamental role in attracting the Symbiodiniaceae cells to establish symbiosis. ActL is a unique coral lectin because it does not attract all Symbiodiniaceae cells, but selectively allows an interaction with only some species, including *Symbiodinium tridacnidorum*, *Symbiodinium tridacnidorum*, *Durusdinium trenchii,* and *Breviolum* sp. The finding that ActL is a main contributor in the acquisition of Symbiodiniaceae cell in the coral *A. tenuis* is valuable, since it supports the role of coral lectins in regulating the attraction and phagocytosis of Symbiodiniaceae cells. This exciting model, supported by strong experimental evidence, encourages us to keep investigating the physiological endogenous role played by lectins in marine organisms.

Wang, Zhou, Liu et al. demonstrate the construction of a oncoVV-WCL recombinant virus, obtained though the insertion of the white-spotted charr lectin (WCL) gene into an oncolytic vaccinia virus (oncoVV) vector [8]. WCL is a rhamnose-binding lectin belonging to the SUEL-type lectin family, identified from white-spotted Charr (*Salvelinus leucomaenis*) eggs based on its primary structure. The viewpoint of this study is quite new, because it is the first to report the combination between a rhamnose-binding lectin and an oncoVV vector. OncoVV-WCL showed an anticancer effect against hepatocellular carcinoma Huh-7cells through activation of caspase-9 and -3. In addition, oncoVV-WCL stimulates the production of IFN-alpha and beta in Huh-7 cells. The anticancer activity of oncoVV-WCL was further demonstrated by inoculating Huh-7 cells into hepatocellular carcinoma tumor-bearing Balb/c nude mice to investigate its function in vivo.

Swarna, Asaduzzaman, Kabir et al. elucidated the diverse immune functions of the sea hare *Aplysia kurodai* egg lectin, AKL-40 [9]. AKL-40 showed cytotoxic activity against human erythroleukemia cells through the activation of signal transduction molecules, but not in human B-lymphoma cells or rat basophilic leukemia cells. The cytotoxic effect was also demonstrated though an in vivo study. AKL-40 was administered to Ehrlich ascites carcinoma cells injected into Swiss albino mice, inducing the reduction of cell viability and cell aggregation. AKL-40 also showed strong antifungal activity against *Talaromyces verruculosus* and antibacterial activity against some bacteria, including *Staphylococcus aureus*. The present study suggests that AKL-40 may contribute to the embryo defense of sea hares.

Grinchenko, Kriegsheim, Shved et al., have reported the biochemical properties of the novel C1q domain containing protein, MkC1qDC, from the hemolymph of the bivalve mollusk *Modiolus kurilensis* [10]. MkC1qDC showed a lectin-like activity by binding galactose and mannose with a requirement of Ca2+. Moreover, it showed antibacterial activity by aggregating both gram positive and negative strains. Among the many compounds tested, alginate, κ-carrageenan, fucoidan, and pectin showed the highest inhibition of MkC1qDC activity. Interestingly, this study implies the potential of biomedical application of MkC1qDC by demonstrating the anticancer effect it exerts on human adenocarcinoma HeLa cells in a dose dependent manner.

Andreeva, Budenkova, Babich et al. report unique and original research which supports the use of carbohydrate additives to increase the biomass and carbohydrate content of cultured microalgae [11]. This finding is highly relevant, since it demonstrates that the nutrient medium can stimulate carbohydrate accumulation in microalgae utilized for biofuel production. In this study, a nutrient medium containing carbohydrate additives was supplied to several species of microalgae, including *Arthrospira platensis*, *Chlorella vulgaris*, and *Dunaliella salina*. The authors have already reported stirring during cultivation as a key factor in reducing carbohydrate production in microalgae. This study provides novel information for improving the culture of microalgae with high carbohydrate content, which may be used for biofuel production.

We are delighted to share these new publications with the scientific community, since we have taken all the necessary steps to ensure their high quality and novelty. We hope that many scientists will enjoy these articles and contribute to start the collaboration with these authors and experts in other scientific areas to develop research fields. Fortunately, the efforts of this Special Issue will be followed by a new Special Issue concerning the same themes, entitled “Marine Glycomics 2.0” (https://www.mdpi.com/journal/marinedrugs/special_issues/marine_glycomics_2, accessed on 12 September 2022).

We invite you to consider submitting manuscripts focused on your recent work for our consideration for a possible publication in this new, upcoming Special Issue. Yuki Fujii, Marco Gerdol, Yasuhiro Ozeki.
